# mRNA, miRNA, lncRNA, ceRNA: O Futuro da Pesquisa Cardiovascular?

**DOI:** 10.36660/abc.20230209

**Published:** 2023-04-20

**Authors:** Gustavo Augusto Ferreira Mota, Mariana Gatto, Cristina Schmitt Gregolin, Sérgio Luiz Borges de Souza, Marina Politi Okoshi

**Affiliations:** 1 Departamento de Clínica Médica Faculdade de Medicina de Botucatu Universidade Estadual Paulista São Paulo SP Brasil Departamento de Clínica Médica – Faculdade de Medicina de Botucatu – Universidade Estadual Paulista (UNESP), São Paulo, SP – Brasil; 2 Departamento de Patologia Faculdade de Medicina de Botucatu Universidade Estadual Paulista São Paulo SP Brasil Departamento de Patologia – Faculdade de Medicina de Botucatu – Universidade Estadual Paulista (UNESP), São Paulo, SP – Brasil

**Keywords:** Epigênese Genética, Remodelação Ventricular, Ácidos Nucleicos, Cardiomegalia, Insuficiência Cardíaca

Apesar do significante avanço na biomedicina cardiovascular, as doenças cardíacas ainda representam marcante problema de saúde pública.^1^ Os indicadores mostram a importância do entendimento dos mecanismos moleculares envolvidos na gênese das doenças cardiovasculares.^2^

Após injúria cardíaca, ocorre o processo de remodelação, definido como alterações genômicas que resultam em modificações moleculares, celulares e intersticiais que se manifestam clinicamente como alterações no tamanho, forma e função do coração.^3,4^ A remodelação cardíaca é, portanto, caracterizada por anormalidades na síntese e degradação de proteínas celulares.

A síntese proteica é dependente dos ácidos ribonucleicos (RNAs). Os RNAs podem ser classificados em RNAs codificantes como o RNA mensageiro (mRNA) e RNAs não codificantes (ncRNAs). De acordo com seu comprimento, os ncRNAs podem ser classificados em ncRNAs longos (lncRNA) e ncRNAs curtos, como os microRNAs (miRNAs). Como o próprio nome diz, os ncRNAs não participam da síntese de proteínas, mas da regulação de RNAs codificantes.^5^

A função de grande número de lncRNAs vem sendo caracterizada.^5^ Os lncRNAs regulam a expressão gênica por mecanismos epigenético, transcricional e pós-transcricional e estão envolvidos no desenvolvimento de hipertrofia miocitária e doenças cardiovasculares.^6,7^ Os miRNAs regulam a expressão gênica degradando ou reprimindo a tradução de moléculas-alvo de mRNA. Assim como os lncRNAs, os miRNAs possuem papel importante na hipertrofia e insuficiência cardíaca.^8^ Denomina-se RNA de competição endógena (ceRNA) o mecanismo regulatório de RNAs pelo qual, por exemplo, um lncRNA pode interagir competitivamente com um miRNA e inibir sua função.

O lncRNA solute carrier family 26 members 4 antisense RNA 1 (SLC26A4-AS1) tem sido associado com hipertrofia cardíaca.^9^ Entretanto, os mecanismos que regulam sua expressão não estão esclarecidos.

Nos Arquivos Brasileiros de Cargiologia, Han et al.,^10^ realizaram ampla investigação sobre o papel do SLC26A4-AS1 na hipertrofia miocitária. Cardiomiócitos ventriculares isolados de camundongos neonatos foram estimulados com angiotensina II (Ang II). O desenvolvimento de hipertrofia foi acompanhado por aumento da expressão do lncRNA SLC26A4-AS1. O fato que a hipertrofia foi atenuada pelo silenciamento do SLC26A4-AS1 sugere relação de causa e efeito entre a expressão do SLC26A4-AS1 e o desenvolvimento da hipertrofia. O silenciamento do SLC26A4-AS1 foi associado a redução da expressão gênica e proteica do solute carrier family 26 member 4 (SLC26A4), mostrando interação entre os dois genes. Finalmente, a Ang II reduziu a expressão do miR301a-3p e miR-301b-3p, e aumento na expressão destes miRNAs suprimiu a hipertrofia induzida por Ang II. O conjunto dos dados permitiu aos autores levantar a hipótese que o SLC26A4-AS1 aumenta a expressão de SLC26A4 e atua como um ceRNA para absorção de miR-301a-3p e miR-301b-3p.

Apesar da extensa metodologia empregada, uma limitação do estudo é o fato que somente experimentos *in vitro* foram realizados. Assim, experimentos *in vivo* serão necessários para confirmar o papel do SLC26A4-AS1 na hipertrofia cardíaca induzida por Ang II.

O estudo mostra a importância do entendimento da rede de interação entre RNAs codificantes e não codificantes na fisiopatologia da hipertrofia miocárdica e sugere o longo caminho a ser desvendado nessa área.
