# Supplementary material for: mRNA, miRNA, lncRNA, ceRNA: O Futuro da Pesquisa Cardiovascular?
Source: Arq Bras Cardiol. 2023 Apr 20;120(4):e20230209. [Article in Portuguese] doi: 10.36660/abc.20230209 (PMC10263425; doi:10.36660/abc.20230209)
Supplement: Translation

# Translation: mRNA, miRNA, lncRNA, ceRNA: The Future of Cardiovascular Research?

**Authors:** Gustavo Augusto Ferreira Mota, Mariana Gatto, Cristina Schmitt Gregolin, Sérgio Luiz Borges de Souza, Marina Politi Okoshi

**Affiliations:** 1 Brazil Departamento de Clínica Médica – Faculdade de Medicina de Botucatu – Universidade Estadual Paulista (UNESP), São Paulo, SP – Brazil; 2 Brazil Departamento de Patologia – Faculdade de Medicina de Botucatu – Universidade Estadual Paulista (UNESP), São Paulo, SP – Brazil

**Keywords:** Epigenesis, Genetic, Ventricular Remodeling, Nucleic Acids, Cardiomegaly, Heart Failure

Despite significant advances in cardiovascular biomedicine, heart diseases still represent a major public health problem.^1^ Thus, it is important to understand the molecular mechanisms involved in the pathophysiology of cardiovascular disease.^2^

Cardiac injury is followed by cardiac remodeling, a process defined as genomic alterations triggering molecular, cellular, and interstitial modifications that manifest clinically as changes in the heart’s size, shape, and function.^3,4^ Therefore, alterations in the synthesis and degradation of cellular proteins characterize cardiac remodeling.

Protein synthesis is dependent on ribonucleic acids (RNAs). RNAs can be divided into coding RNAs, such as messenger RNA (mRNA), and non-coding RNAs (ncRNAs). According to their length, ncRNAs can be classified into long ncRNAs (lncRNA) and short ncRNAs such as microRNAs (miRNAs). As the name indicates, ncRNAs are not involved in protein synthesis but in modulating coding RNAs.^5^

The function of a large number of lncRNAs has been characterized^5^. lncRNAs regulate gene expression by epigenetic, transcriptional, and post-transcriptional mechanisms and are involved in developing myocyte hypertrophy and cardiovascular diseases.^6,7^ miRNAs regulate gene expression by degrading or repressing the translation of target mRNA molecules. Like lncRNAs, miRNAs play an important role in hypertrophy and heart failure.^8^ Endogenous competition RNA (ceRNA) is a regulatory mechanism of RNAs that permits, for example, an lncRNA to competitively interact with a miRNA and inhibit its function.

The lncRNA solute carrier family 26 members 4 antisense RNA 1 (SLC26A4-AS1) has been associated with cardiac hypertrophy.^9^ However, the mechanisms regulating its expression are not clear.

In the of *Arquivos Brasileiros de Cardiologia*, Han et al.^10^ performed extensive research on the role of SLC26A4-AS1 in myocyte hypertrophy. Ventricular cardiomyocytes isolated from neonatal mice were stimulated with angiotensin II (Ang II). The development of hypertrophy was associated with increased expression of lncRNA SLC26A4-AS1. The fact that hypertrophy was attenuated by silencing SLC26A4-AS1 suggests a cause-and-effect relationship between SLC26A4-AS1 expression and hypertrophy development. SLC26A4-AS1 silencing was accompanied by a reduced gene and protein expression of the solute carrier family 26 member 4 (SLC26A4), showing an interaction between the two genes. Finally, Ang II reduced miR301a-3p and miR-301b-3p expression, and the increase in expression of these miRNAs suppressed Ang II-induced hypertrophy. The data allowed the authors to hypothesize that SLC26A4-AS1 increases SLC26A4 expression and acts as a ceRNA for sponging miR-301a-3p and miR-301b-3p.

Despite the extensive methodology employed, the study’s limitation is that only *in vitro* experiments were performed. Thus, *in vivo* experiments will be needed to confirm the role of SLC26A4-AS1 in Ang II-induced cardiac hypertrophy.

The study shows the importance of understanding the interaction network between coding and non-coding RNAs in the pathophysiology of myocardial hypertrophy and suggests a long way to go in this area.
